# How can healthcare organisations improve the social determinants of health for their local communities? Findings from realist-informed case studies among secondary healthcare organisations in England

**DOI:** 10.1136/bmjopen-2024-085398

**Published:** 2024-07-25

**Authors:** Anna Gkiouleka, Luke Munford, Sam Khavandi, Ruth Watkinson, John Ford

**Affiliations:** 1 Wolfson Institute of Population Health, Queen Mary University of London, London, UK; 2 School of Health Sciences, The University of Manchester, Manchester, UK; 3 NIHR Applied Research Collaboration Greater Manchester (ARC-GM), Manchester, UK

**Keywords:** health services, health equity, health policy, organisation of health services, hospitals

## Abstract

**Objectives:**

Increasingly, healthcare and public health strategists invite us to look at healthcare organisations as not just care providers but as anchor institutions (ie, large community-rooted organisations with significant impact in the local economy, social fabric and overall community well-being). In response, this study explores the mechanisms through which healthcare organisations can impact social determinants of health and communities in their local areas.

**Design:**

We conducted case studies with interviews and synthesised the findings using a realist approach to produce a set of explanations (programme theory) of how healthcare organisations can have a positive impact on the overall well-being of local communities by operating as anchor institutions.

**Setting:**

Secondary healthcare organisations in England, including mental health and community services.

**Participants:**

Staff from case study sites which were directly employed or actively engaged in the organisation’s anchor institution strategy. Data collection took place from early June to the end of August 2023.

**Results:**

We found four building blocks for effective anchor activity including employment, spending, estates and sustainability. Healthcare organisations—as anchor institutions—can improve the social determinants of health for their local communities through enabling accessible paths for local community recruitment and career progression; empowering local businesses to join supply chains boosting income and wealth; transforming organisational spaces into community assets; and supporting local innovation and technology to achieve their sustainability goals. These blocks need to be integrated across organisations on the basis of a population health approach promoted by supportive leadership, and in collaboration with a diverse range of local partners.

**Conclusions:**

Healthcare organisations have the potential for a positive impact on the overall well-being of local communities. Policymakers should support healthcare organisations to leverage employment, spending, estates and sustainability to help address the unequal distribution of the social determinants of health.

**STRENGTHS AND LIMITATIONS OF THIS STUDY**

- This realist-informed study offers significant insights to the underlying mechanisms through which healthcare organisations can have an impact on the social determinants of health in their local communities.
- We included a diverse range of case studies that differ in terms of offered services, anchor activity strategy, size and areas, which ensures the transferability of the results.
- Due to the current lack of bespoke quantitative data, the study results do not offer measurable indicators of anchor activities, but rather an overall theory of how anchor institutions may affect the local economy and communities.

## Introduction

 Healthcare organisations aim for community health by offering treatment and prevention services. However, decades of research shows that health results from the conditions in which people are born, grow, live and work; the so-called social determinants of health.^1^ Inequalities in these conditions (eg, housing, income) have a disproportionate burden of morbidity and mortality for individuals and groups experiencing social and economic disadvantage.^2^

Lately, healthcare and public health strategists highlight the evolving role of healthcare organisations as not just care providers but as economic players with significant assets and job creation capacity.^3^ Healthcare organisations can significantly impact local economies improving social determinants of health, and thereby promoting better population health and reducing health inequalities.^4^ Because of this impact, healthcare organisations emerge as anchor institutions—large organisations rooted in specific areas and communities, using substantial resources to address social needs and enhance community well-being.^3^,^5^

For example, in the UK, the National Health Service (NHS) is the biggest employer in the country employing over 1.36 million people, excluding primary care staff.^6^ Especially in socioeconomically disadvantaged regions, the role of the NHS as a major employer is proliferated with healthcare organisations representing a vital part of local economic activity.^6 7^ It can narrow socioeconomic and health inequalities by offering employment opportunities for people facing barriers to entering the labour market (eg, long-term unemployed, people with limited marketable skills) and by being an exemplary and environmentally conscious employer.^4 5^ Further, through procurement and commissioning, it can sustain inclusive economic growth and positively impact local communities by increasing access to quality housing, health promoting infrastructure and leisure spaces.^3 8^ NHS estates portfolio covers roughly 6500 hectares of land including buildings and their surrounding physical environment.^9^ Leveraging this capital, collaborating with partners and ensuring environmental sustainability, the NHS can function as an economic development engine, linked to healthier communities and more equity in social and health outcomes.^10^,^12^

The COVID-19 pandemic exacerbated inequalities across health and overall economic prosperity in many countries.^13^ In response, health systems are being redesigned to integrate health and social care, local government and public health to support inclusive social and economic development and reduce health inequalities.^14^ In this effort, fostering effective anchor-related activities especially in socioeconomically disadvantaged areas is necessary. Currently, there is limited evidence on how healthcare organisations can effectively act as economic players to improve the well-being of local communities. This study investigates the mechanisms through which healthcare organisations—as anchor institutions—can impact social determinants of health for their local communities.

## Methods

### Study design

Our research team comprised researchers from various backgrounds (ie, health economics, public health, sociology, community engagement), balanced in terms of gender and with different ethnic backgrounds. Following previous research, we undertook case studies informed by a realist approach.^15^ Realist approaches focus on the identification of mechanisms operating in specific contexts and resulting in specific outcomes, to develop a programme theory of what works, for whom, in what circumstances and how.^16 17^ As per realist methodology, we started by developing a broad explanation (ie, initial programme theory) of how secondary healthcare organisations can operate as anchor institutions and impact their local economies. We did this by reading peer reviewed and grey literature, discussing within the team and receiving experts’ feedback.^18^ Our initial programme theory suggested six main ways in which healthcare organisations can achieve this: (1) budget spending, (2) employment opportunities, (3) land and building use, (4) environmental impact, (5) promoting social well-being and (6) major restructures (eg, service openings or closures). The initial programme theory informed our topic guide, which we then used to collect data to populate each of these six domains and refine our explanation. We created our topic guide through discussions within the team so that it included questions addressing each of the domains identified in the initial programme theory and relevant prompts. We shared a first draft with our group of patient representatives and integrated their feedback in a final version (available in the online supplemental material). Through data collection and synthesis, we refined the programme theory to derive robust and transferrable conclusions on how healthcare organisations can bolster local economies.

### Recruitment of case study sites

For the selection of case study sites, we used an analysis of all the secondary healthcare organisations in England, including mental health and community services. The analysis included clinical to non-clinical staff ratios, the total amount collected by local authorities based on Trust Accounts Consolidation data,^19^ the total non-clinical space in square metres based on Estates Return Information Collection data^20^ and the percentage of total spending across a 10 km radius around a healthcare organisation as a proxy for an ‘anchor area’, based on NHS procurement data.^21^ Organisations were ranked from lowest to highest score across all measures and the analysis provided us with the largest drop and increase in rank across 4 years (2017–2021). For each measure, we chose the top and bottom 10 sites. We combined the top 10 sites into a highest ranking list and the bottom 10 into a lowest ranking list across all measures. Then, we identified sites that appeared multiple times in the highest or lowest ranking list.

We conducted desktop research on anchor-related activities (ie, initiatives within a set anchor institution strategy or social value programmes) in each shortlisted site, choosing four illustrative case studies—two from the highest and two from the lowest ranking list. Finally, we chose sites located in socioeconomically disadvantaged areas which were diverse in terms of size, geography, services and anchor activity approach. This data driven approach ensured the non-biased selection of case studies from a pool of healthcare organisations that were diverse across a series of comparable objectively measured indicators. Further, we focused on socioeconomically disadvantaged areas because healthcare organisations in those areas serve the people most severely affected by health inequalities. We considered that this might imply a greater engagement with anchor activity but also challenges resulting from the increased patient need. This ensured that our findings are meaningful within the current challenging circumstances in the healthcare system.

### Recruitment of participants and data collection

The medical director of each site disseminated recruitment material among staff who had knowledge about the organisation’s anchor-related activities (eg, health inequalities lead, procurement team, human resources). Interested individuals received a participant information sheet and a consent form via email. Those who agreed to participate returned the form completed and signed via email before the beginning of data collection. Participants were not compensated for their time.

Data collection took place from early June to the end of August 2023. It involved one-to-one interviews or small group discussions based on the preagreed topic guide. Sessions were conducted in-person or online according to participants’ preferences. Based on realist methodology,^22^ AG started every session by presenting some broad conclusions about the organisation’s anchor institution activity (derived through desktop research) to keep the discussion close to facts rather than participants’ perceptions. Sessions lasted between 30 and 90 min and were audio recorded. None of the team members had previous relationships with the research participants.

### Data analysis

Audio records were anonymised and transcribed by a UK transcription service after signing a data protection agreement. The transcripts were uploaded in Nvivo and coded by AG. The codes were either decided in advance of the analysis based on the initial programme theory (*deductive*), for example, types of employment in the organisation. Alternatively, they were created during the analysis to categorise data found in the transcripts (*inductive*), for example, staff well-being. In some cases, they were created on the base of data interpretations about the possible mechanisms leading to observed outcomes (*retroductive*), for example, shared learning.^17^ AG organised the codes in themes and identified causal statements (*CMOC configurations*) about a certain Context (C) activating a certain Mechanism (M) leading to a certain Outcome (O) across themes (table 1). To enhance trustworthiness, themes were discussed and refined within the team and in consultation with our Public & Community Involvement & Engagement group (PCIE) described in the next section.

**Table 1:** Context–Mechanism–Outcome configurations

Context	Mechanism	Outcome
Alternate recruitment paths, technical support and well-being projects for staff.	Organisations become more visible as potential attractive employers for local disadvantaged communities.	Widened participation of disadvantaged communities in recruitment and employment programmes and workforce retention.
Knowledge of local markets and provision of technical support to communities.	Identification of economic opportunities and enablement of local suppliers to engage in bids and competitions.	Increased income and potentially wealth for local businesses and employees.
Open buildings and events that enable staff and community exchanges.	Community engagement.	Improved staff well-being and increased income for local farmers or sellers.
Investment in innovation and technology to meet climate targets engaging local communities.	Opportunities for employment and economic activity for local businesses and communities.	Increase in local businesses’ capacity, income and potentially wealth.
Adopting a population health approach with supportive senior leadership, agreed targets and regular performance monitoring.	Integration of anchor activities across the organisation and tailoring to local needs.	Greater staff and community engagement with anchor activity and social value projects.
Place-based equal partnerships with other anchor organisations and experienced community engagement actors.	Trusting relationships with local actors and communities and cocreation of anchor projects.	Widened participation of local communities in anchor activity and maximisation of impact.

### Patient and public involvement

We worked closely with a diverse PCIE from the Greater Manchester (GM) community. We recruited participants through our partnership with National Institute for Health and Care Research Applied Research Collaboration for GM^23^ Public and Community Involvement, Engagement, and Participation group and the respective Young People Advisory Research group. Our PCIE group comprised five individuals with a diverse range of experience with healthcare services and community work. In their majority, members of our group were people from ethnic minority backgrounds. RW, AG and SK were the main points of contact for public involvement. We held five online meetings across different stages of the study where our contributors shared insights and feedback on: the formulation of research questions, quantitatively measuring anchor activity, selection of case studies, development of interview topic guide, findings and conclusions. In addition, conversations were held on a one-to-one basis via email or online meetings. All the members of our PCIE group were reimbursed for the time they engaged with our research.

## Results

In total, we recruited 22 participants from four sites. The chosen sites offered a diverse range of services including community, mental health and hospital services. They varied in terms of annual income ranging from less than 100 million to more than 1500 million pounds and workforce size ranging from less than 5000 to more than 20 000 employees. The participants included clinical and non-clinical employees at different seniority levels and external partners engaged in the organisation’s anchor activity. Information on participants per site are available in table 2.

**Table 2:** Study sites and participants

Case	Services	Annual income	Staff	Region	Roles
C1	Hospital services	>1500 million	>15 000	North West	Director of Social Value Creation (1)Chief of People (1)Healthcare scientist (1)Social Value Lead (1)Head of Sustainability (1)Place director (1)External partner (2)CEO infrastructure organisations (1)Public health registrar (1)Career ambassadors (2)
C2	Hospital services	>1000 million	>10 000	North East	Director of operators (1)Charity director (1)Clinical lead (paediatrics) (1)Assistant chief executive (1)Head of sustainability (1)
C3	Mental health and community services	>500 million	>5000	Greater London	Head of resourcing (1)Public Health Registrar (1)Associate Director of Contracts & Procurement (1)Deputy Chief Executive (1)
C4	Community services	<100 million	<5000	North West	Inclusion, Diversity & Health Inequalities Lead (1)

**Figure 1:**
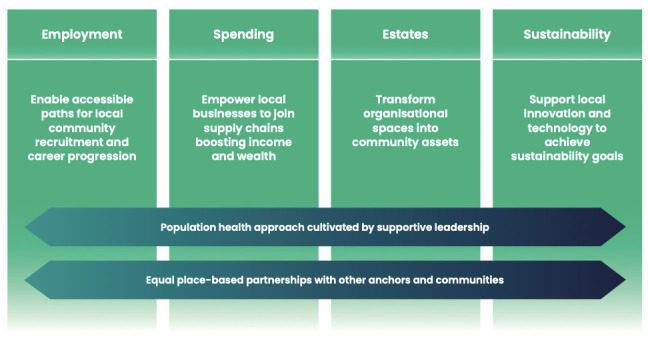
The four blocks of effective anchor activity.

Our findings showed that a framework for effective anchor activity includes four building blocks; namely, employment, spending, estates and sustainability, held together by organisational ethos based on a population health approach and local partnerships (figure 1). Further details on the framework for effective anchor activity are discussed below with illustrative data excerpts.

### Employment

Participants shared an interest in widening local community participation in recruitment, employment programmes and future workforce. However, organisations had different strategies to create employment opportunities for residents in their areas. In two sites, the impact was more visible in the short-term. Those sites had developed alternative recruitment paths based on direct interaction between candidates and recruiting staff. This involved organising career events in community spaces and promoting opportunities through multiple channels. As the excerpts below show, such initiatives were particularly effective in reaching disadvantaged people who are often discouraged by online applications.

In deprived neighbourhoods [ … ] their ability to answer… read and answer all the questions on forms, is tricky. And we know that from anecdotal feedback at the recruitment fares, people give up the ghost when they’re applying for an NHS job, because it’s just too hard. C1.10

The basic NHS employer’s check standards stipulates we need to have this, this [ … ] to enable this person to start. [ … ] It’s quite an exhaustive list and hence a time to hire. Some people lose interest. [ … ] It’s not as much about getting our communities just interested in working with us, but it’s also how do we retain that interest and get them in quickly. C3.1

Alternative recruitment paths paired with consistent support for candidates and new employees made these organisations more visible as attractive employers in their area. The outcome was an increase in the number of people from local disadvantaged communities among the organisation’s staff and improved staff retention as shown below.

I’ve attended career events or jobs fairs with widening participation [ … ] making people aware about the varieties of roles available, ‘cause I think often people think about the obvious, doctors, nurses [ … ] they don’t think about all the other jobs that go on to make the hospital and the services run. C1.7

We interview them and then they fill the form in, so we do that the other way around. And we’ve recruited [ … ] 80 people from one of them. In a day recruiting 80 people is good, isn’t it? C1.10

### Spending

Participants commonly believed that achieving local impact through procurement and spending is often complicated by bureaucracies in national supply chains or the lack of competitiveness among local suppliers compared with national ones.

Nationally, the national drivers contradict social value. There is a national push for the NHS to buy in bulk [ … ] most things we buy are on a national framework. Now, the national framework is guidance but if you don’t do it when you’re in financial trouble, the first thing they’ll do is say (you) failed as an organisation because you didn’t follow the cheap route. C1.3

There was a risk associated with having social value weighted quite highly, I think it’s 25% for all our contracts. There was a risk for small and medium sized businesses and potentially local businesses as well, that that would be a barrier to them bidding for contracts. Because it would be unfamiliar and outside their area of expertise and they would struggle to make a case. C3.2

In two of the studied sites, procurement teams sought to understand their local markets and provided technical support to local businesses regarding NHS frameworks and tendering. These context elements revealed opportunities for healthcare organisations to choose existing local suppliers for the provision of necessary services without undertaking extra costs.

My work is with procurement teams and helping them to understand in terms of how they choose suppliers, where they get suppliers, what’s important to them when choosing suppliers and helping them to think about the impact procurement could have in terms of reducing health inequalities. C1.2

So we did a piece of work to develop a social values toolkit [ … ] Which is as it says the kind of guidance for those smaller organisations on how to go through the process. C3.2

Further, the provided support facilitated increased participation of local suppliers in bids and competitions. While not immediately measurable, the outcome is potentially an increase in income and wealth generation for local businesses and their employees.

We tend to talk about wealth not income because it’s about control and ownership [ … ] not just how can we enable places [ … ] to spend more money locally with any business, and that’s okay but if we can help them to understand alternative business models like cooperatives [ … ] more of that money will stay in low income communities but also, those communities will for the first time in their lives [ … ] own something and we know that improves health. C1.1

### Estates

Participants discussed the utilisation of health organisations’ spaces to host local businesses, services or community events as a pathway for effective anchor activity. A common strategy across the sites concerned hosting food markets with affordable local produce on the organisations’ premises. Such initiatives strengthened community engagement and benefitted both organisation staff with access to affordable healthy food and local farmers and sellers with increased income.

we have food and vegetable stalls, which obviously have a number of different beneficial effects, that allows the staff to buy fresh fruit and vegetables onsite [ … ] and obviously the people who are running those fruit and vegetable stalls, are local [ … ] so they are getting the financial benefits of that. C2.2

As the following excerpt shows, other effective use of spaces included offering rooms and facilities necessary for charities and local organisations to provide their services.

We’ve got quite a few initiatives where we’re sharing our spaces with other organisations. We’re a mental health trust primarily so we’ve got at least three places where the charity [ … ] are co-located. So, they can be right there where people are already accessing services in order to reach them. C3.2

Understanding local needs and maintaining open communication channels with communities was key for keeping the organisations’ doors open to local people.

Because what we need to do is match it up with somebody who wants that opportunity and needs that thing, and for them to practically work (it) out. C1.8

### Sustainability

Healthcare organisations in the UK are committed according to national guidelines to minimise their carbon footprint. This opens up an additional channel through which they can positively impact their local communities. By eliminating their carbon footprint, healthcare organisations will relieve their local areas from a source of pollution with associated health benefit. As demonstrated below, meeting climate targets requires organisations to invest in innovation and technology. This can create employment opportunities, increase knowledge, income and wealth for local businesses and amplify the impact of sustainability interventions.

What we've tried to do in terms of procurement and opening the doors to local innovation and local business is really spreading out when we start talking about sustainability [ … ] we've really split it down in plain English [ … ] so from a window cleaning perspective, how are you looking at utilising the use of detergents and the use of water? What technology are you going to be using? C1.3

By us working with more and more businesses and supporting the population that we have, and the businesses that are surrounding us we’re actually in the grander scheme of things becoming a sustainable (place) C1.3

Adapting old buildings and infrastructure for greater energy efficiency or waste management are areas where local innovation can prove beneficial and yield practical advantages for the everyday experience and overall well-being of local communities.

We’re on the theme of land and buildings trying to work to green more space and become connectors for things like green corridors [ … ] volunteering space in our estates to contribute to local initiatives [ … ] we can provide things like secure cycle storage and electric vehicle charging points. Which although they would be within the footprint of our land would also be accessible to people in our communities. C3.2

### Organisational ethos based on a population health approach

When healthcare organisations embrace a population health approach with agreed targets and ongoing performance monitoring, anchor activity becomes integrated across the organisation. When led by people who appreciate the importance of strengthening communities and social value, due to lived experience, related projects are better tailored to local needs. The outcome is increased and more consistent engagement by both organisation’s staff and community members with anchor activity. For example, a participant highlighted how adopting a population health perspective is a way an organisation can help the communities it serves to thrive.

What I can share with us is the kind of statement that we’ve agreed around trying to explain what we’re doing in terms of our population health work. [ … ] We don’t see ourselves merely as a health care provider, we have a responsibility to do our bit to make our corner of the world a fairer place to live and work. We want to see the communities we serve thrive. Our work on population health [ … ] is our way of turning this aspiration into reality. C3.2

And added that this is easy to explain to the organisation’s staff because they know how life conditions affect health.

[ … ] clinicians often know and are really concerned about the social determinants of their patients’ health. They can see that part of the reason for people’s difficulties is their terrible housing or the fact that they can't find good work. C3.2

What seems to make the difference is having supportive leaders who understand the importance of social value due to their own lived experience. The excerpts below demonstrate these points.

You need to have your executive directors, and non-executive directors to be completely on board with this approach and strategy and that’s really helped us. C3.3

Equality and diversity is a really important one for us. [ … ] We're doing a lot of work around that to become as open and as accepting an organisation as we can be. [ … ] I think perhaps the first thing that people saw was us talking about sexuality and pride and we had a pride flag raised very soon after the chief exec started. She’s a gay woman, we've got a couple of other people on the senior management team that are gay and that are very comfortable to talk about that. We've made a huge fuss around pride and really brought that into the organisation. C2.1

### Local partnerships

Anchor activity is more effective when healthcare organisations participate in place-based partnerships with other anchor organisations, such as universities, housing associations and experienced community engagement actors. Key to these partnerships is that healthcare organisations join as equal members contributing resources and technical expertise. Being a visible and ‘humble’ partner builds trust with local communities creating opportunities for cocreated projects. This results in increased community engagement in anchor activity and maximises impact as illustrated in the excerpts below.

we’ve developed a [ … ] network in which the eight local anchors come together [ … ] to work on these things jointly in terms of permanent spend [ … ] priorities that they have, how they can do that on a more collective basis [ … ] Partnership creates more resources but partly because it brings all those different mindsets together. C1.1

We’ve got the jobs, we need someone who can do the employability, now that sometimes is us, sometimes it’s someone else and then we need somebody else who can reach the communities and do the pastoral care and sometimes that isn’t us because we’re not that good at it. C1.4

## Discussion

### Principal findings

Our realist case studies revealed that healthcare organisations—as anchor institutions—can improve the social determinants of health of local communities using four building blocks: (1) employment: enabling accessible paths for local community recruitment and career progression; (2) spending: empowering local businesses to join supply chains boosting income and wealth; (3) estates: transforming organisational spaces into community assets; and (4) sustainability: supporting local innovation and technology to achieve their sustainability goals. To be cohesive, this set of building blocks requires an organisational ethos based on a population health approach promoted by supportive leadership, and close collaboration with a diverse range of local partners including other anchor institutions and community engagement actors.

### Study strengths and weaknesses

Our study’s strengths lie in its methodology and diverse case studies. Realist methodology enabled us to understand how things work and identify facilitators and barriers that healthcare organisations encounter in developing their anchor activity. Further, it helped us combine factual evidence with the knowledge of experienced people in the field. This provided us with unique insights to the challenges around anchor activity, which may not have been included in official reports or other documents. Further, the diversity of studied sites helped us explore different anchor activity paradigms and identify commonalities and transferrable lessons, which then informed our anchor activity framework.

Our study draws on system redesign and organisational transformation in a UK setting, and therefore may have limited generalisability in healthcare settings with different levers for organisational change. The main study weakness concerns the current lack of publicly available measurable and comparable data on the scale and impacts of anchor activity. Participants noted that anchor activity is a long-term project with long-term effects, requiring substantial resources for monitoring at the community or regional level. At the time of the study, none of the participating organisations had such data. However, a realist analysis focuses on the mechanisms of impact rather than impact size.^17^ Therefore, we are confident that our conclusions are robust and meaningful for researchers and practitioners, highlighting the potential that healthcare organisations have to operate as anchor institutions.

### Findings in the context of previous work

Consistent with recent findings,^4 8 24^ our study suggests that anchor activity should be organised across four different blocks including employment, spending, estates and sustainability. We also found that to positively impact their communities, healthcare organisations need to actively engage in local partnerships with a diversity of partners including other local anchors such as universities and experienced community engagement actors.^8^ Such findings agree also with global health literature on the importance of local health committees in the creation of responsive health systems.^25 26^ Our study adds that these partnerships should be founded on equality and shared goals. It underscores the importance of organisational leadership, noting that impactful leaders are those who understand the importance of strengthening communities, often due to their lived experience, and promote a population health approach across the organisation. Diverging slightly from previous frameworks, we suggest that organisational ethos and local partnerships should be understood not as separate areas of activity but rather as the connecting material that keeps anchor activities integrated across the organisation.

### Implication for future research, practitioners and decision makers

Our study highlights the breadth of anchor activities that healthcare organisations undertake. Policymakers should facilitate effective partnerships between healthcare organisations and local community groups, support the procurement of cost-effective services which support the local economy, optimise the use of estates and use employment practices benefiting local people.^8^ Policymakers should acknowledge that overburdening local healthcare organisations with national directives can hinder organisations’ ability to fulfil ambitions such as becoming anchor institutions. Supported by national policymakers, healthcare organisations should develop their own metrics to assess their performance according to the building blocks described in this study. This requires senior leadership engagement and cultivating an ethos which supports the social determinants of health. Individuals with lived experience of disadvantage who are committed and motivated can and should be enabled to partner in organisational change. Further research should assess the quantitative benefits of anchor activities, focusing on factors that yield the highest impact for local areas. Existing measurement toolkits^24^ are already offering a range of useful indicators for measuring this impact. Future research can help us elaborate and refine such toolkits. Additionally, research is needed on effective leadership and partnership models for anchor activity that integrates the views of local communities on the impact of healthcare organisations on their own life circumstances.

## Conclusion

Healthcare organisations can positively impact local communities through employment, spending, estates and sustainability initiatives, employing a population health approach in their organisational ethos and being equal members of place-based partnerships with other anchors and communities.

## supplementary material

10.1136/bmjopen-2024-085398 online supplemental file 1

## Data Availability

Data are available on reasonable request.
