# Supplementary material for: How can healthcare organisations improve the social determinants of health for their local communities? Findings from realist-informed case studies among secondary healthcare organisations in England
Source: BMJ Open. 2024 Jul 25;14(7):e085398. doi: 10.1136/bmjopen-2024-085398 (PMC11284868; doi:10.1136/bmjopen-2024-085398)
Supplement: online supplemental file 1 [file bmjopen-14-7-s001.pdf]

A. Introductions

B. Aim of the discussion: Today we would like to discuss whether and how the hospital (details of the hospital) affects the local area and its communities in terms of economic activity and to what extent this can improve people's health and reduce health inequalities.

Details: The discussion will last between 30 and 60/90 minutes but you are free to have a pause or withdraw at any point. Anything discussed is confidential, so you are kindly asked to not share details about the content of the discussion or the other participants with other people. The information you will share will be completely anonymised and we will make sure that there is no possibility for you to be identified at any stage of the research process.

Please share your thoughts and opinions as honestly as possible. If you wish to give examples, please do not mention anything that might be identifiable. You are kindly requested to use respectful language and make sure that everybody has the time to share their input.

Consent for audio recording.

C. Questions

Introduce the participant(s) in the topic of the discussion and give some examples of anchor activity from the data.

1. Do you think that the hospital spends its budget in a way that benefits the local market?
Prompt: healthcare products/services/food
Prompt: barriers/facilitators

2. Do you think that the hospital supports the residents of the area to get a job?
Prompt: direct employment vs contracts/roles/demographics/training
Prompt: barriers/facilitators

3. Do you think that the local residents and the community benefit from the hospital's property (buildings, land etc)?
Prompt: housing/community spaces/ green spaces/job openings for construction companies and skilled workers
Prompt: barriers/facilitators

4. How would you describe the impact of the hospital on the physical environment of the area?
Prompt: pollution/recycling/traffic/food waste

5. Would you say that the hospital contributes to the overall social wellbeing of the area and its people?

Prompt: collaborations and partnerships/ setting a good example /improve people's living conditions
Prompt: barriers/facilitators

6. Do you think that after the hospital opened the residents of the area have better living conditions? Has this affected their health?
OR
Do you think that after the hospital closed down the residents of the area have worse living conditions? Has this affected their health?
Prompts: work/income/physical environment/neighbourhoods/ groups affected
Prompt: most important reason that this happened
