# Supplementary material for: How can healthcare organisations improve the social determinants of health for their local communities? Findings from realist-informed case studies among secondary healthcare organisations in England
Source: BMJ Open. 2024 Jul 25;14(7):e085398. doi: 10.1136/bmjopen-2024-085398 (PMC11284868; doi:10.1136/bmjopen-2024-085398)
Supplement: Supplementary file 2 [file bmjopen-2024-085398.reviewer_comments.pdf]

PEER REVIEW HISTORY

BMJ Open publishes all reviews undertaken for accepted manuscripts. Reviewers are asked to complete a checklist review form (**http://bmjopen.bmj.com/site/about/resources/checklist.pdf**) and are provided with free text boxes to elaborate on their assessment. These free text comments are reproduced below.

ARTICLE DETAILS

**TITLE (PROVISIONAL)**	How can health care organisations improve the social determinants of health for their local communities? Findings from realist-informed case studies among secondary health care organisations in England
**AUTHORS**	Gkiouleka, Anna; Munford, Luke; Khavandi, Sam; Watkinson, Ruth Elizabeth; Ford, John

VERSION 1 – REVIEW

**REVIEWER**	Bludau, Heidi<br>Vanderbilt University, Medicine, Health, and Society
**REVIEW RETURNED**	01-Mar-2024

**GENERAL COMMENTS**	You state that you developed a programme theory but you do not discuss that theory or if you do it is unclear that you discuss it. What is the programme theory developed which in turn informed your research instruments?

**REVIEWER**	Ramirez, Paulina<br>University of Birmingham, Business School
**REVIEW RETURNED**	08-Mar-2024

**GENERAL COMMENTS**	This is an interesting study that raises important issues given existing health inequalities.<br><br>Given the lack of data and the fact that these are long term initiatives that have yet to be assessed, the emphasis of the paper should be one of “potential” rather than actual benefit. The article could be made more useful if there was a table suggesting how impact could be assessed in future.<br><br>The objective of the study are rather vague at the beginning of the paper (page 4, line 50) and in the abstract. The objective- a study of the mechanisms by which health care organisations can impact social determinants and local communities- should be made clearer in the abstract, in the introduction and throughout the paper.<br><br>A more critical engagement with the literature on the processes of health care re-design is also needed. The present processes of transformation of the UK health care system have been interpreted in different ways and it is important to acknowledge the contested nature of these changes. The interpretation of the present changes adopted by the study is one amongst many and this should be acknowledge.

	More detail of the data-driven approach to the selection of the case studies is needed. What did this approach contribute to the analysis and how are the cases chosen by this method related to the case studies located in areas of social and economic disadvantage.<br><br>The article refers to the programme theory. Please explain this in more detail and how this helped the decision of the coding.<br><br>More detail is also necessary on the section on patient and public involvement. How many public discussions groups?, what were their composition? Where any community organisations involved? If so who did they represent? Given the poor mechanisms for public and patient involvement in the health care sector, more detail of the scale and nature of this participation is needed.

**REVIEWER**	Reyes-Santías, Francisco<br>Universidad de Vigo, Organización de Empresas e Mercadotecnia
**REVIEW RETURNED**	11-Mar-2024

**GENERAL COMMENTS**	The paper "How can health care organisations improve the social determinants of health for their local communities: Realist informed case-studies", performs a study that explore how health organisations can act effectively as grassroots institutions to improve the social determinants of health for people in their communities.<br><br>Title and summary. The title and abstract express well the object of study, objectives, and results of the article.<br><br>Structure of the article. The contents are well organized and they adhere to the IMRaD structure. It includes a theoretical framework of the research problem, but at this point, I suggest the authors incorporate some other bibliographic references that I miss in the text:<br><br>Mulumba M, London L, Nantaba J, Ngwena C. Using Health Committees to Promote Community Participation as a Social Determinant of the Right to Health: Lessons from Uganda and South Africa. Health Hum Rights. 2018 Dec;20(2):11-17. PMID: 30568398; PMCID: PMC6293345.<br><br>Ruano AL, Friedman EA, Hill PS. Health, equity and the post-2015 agenda: raising the voices of marginalized communities. Int J Equity Health. 2014 Oct 10;13:82. doi: 10.1186/s12939-014-0082-6. PMID: 25300905; PMCID: PMC4201725.<br><br>Focusing on the opportunity of the study, it must be said that it is useful work since it covers one of the major problems resulting from a health care models.<br><br>Materials and methods.<br>Regarding the material and methods section, the methodology is tailored to the object of study and the objectives and is explained in a transparent manner while it has been validly applied to guarantee the results.<br><br>Results.<br>The results are significant and they are presented in an adequate and understandable way not only through narration but also with

	self-explained tables that are also well elaborated in terms of presentation. The results justify and relate to the objectives and methods and the results are of sufficient interest.<br><br>Discussion<br>It would be interesting to introduce in the paper a discussion to appropriately compares the study results with other works, highlighting the main study findings. I would suggest the inclusion of three bibliographic references in the discussion section:<br><br>Ahmed, S., Chase, L.E., Wagnild, J. et al. Community health workers and health equity in low- and middle-income countries: systematic review and recommendations for policy and practice. Int J Equity Health 21, 49 (2022). https://doi.org/10.1186/s12939-021-01615-y<br>Andermann, A. Screening for social determinants of health in clinical care: moving from the margins to the mainstream. Public Health Rev 39, 19 (2018). https://doi.org/10.1186/s40985-018-0094-7.<br><br>Bibliography<br>The 66.6% of the bibliography cited in the study belongs to the previous five years.<br><br>Overall, it is an interesting study and should be considered for publication in BMJ Open, once the minor revisions proposed have been resolved.

**REVIEWER**	Mateus, Ceu<br>Lancaster University, Division of Health Research
**REVIEW RETURNED**	13-Mar-2024

**GENERAL COMMENTS**	This is an interesting paper resulting from informed case studies. the capacity for health care organisations to improve the social determinants of health for their local communities seem to exist, it would be interesting to see how it materializes. It's interesting that the working culture of the institutions was not considered as relevant for the development of anchor activities by the institutions. It would be relevant to have some demographic characteristics of the sample like gender split, mean age, and number of years in the role.

VERSION 1 – AUTHOR RESPONSE

Reviewer: 2
Dr. Heidi Bludau, Vanderbilt University
Comments to the Author:
You state that you developed a programme theory but you do not discuss that theory or if you do it is unclear that you discuss it. What is the programme theory developed which in turn informed your research instruments?
• Thank you for your comment. We have elaborated the study design section to address this gap by adding the following text on p.5, lines 8-21.
"As per realist methodology, we started by developing a broad explanation (i.e., initial programme theory) of how secondary healthcare organisations can operate as anchor institutions and impact their local economies. We did this by reading peer reviewed and grey literature, discussing within the team, and receiving experts' feedback.18 Our initial programme theory suggested six main ways in which

health care organisations can achieve this: i) budget spending ii) employment opportunities iii) land and building use iv) environmental impact v) promoting social well-being vi) major restructures (e.g., service openings or closures). The initial programme theory informed our topic guide which we used to collect data to populate each of these six domains and refine our explanation. We created our topic guide through discussions within the team so that it included questions addressing each of the domains identified in the initial programme theory and relevant prompts. We shared a first draft with our group of patient representatives and integrated their feedback in a final version (available in the supplementary material)."

Reviewer: 3
Dr. Paulina Ramirez, University of Birmingham
Comments to the Author:
This is an interesting study that raises important issues given existing health inequalities.

Given the lack of data and the fact that these are long term initiatives that have yet to be assessed, the emphasis of the paper should be one of "potential" rather than actual benefit.
• Thank you for your comment. Indeed, with our article we aim to show how health care organisation can impact their communities. This is why we 've chosen phrases like "This study investigates the mechanisms through which health care organisations -as anchor institutions- can impact social determinants of health for their local communities." Which is also the way we have formulated our title. Our case studies show that healthcare organisations to a different degree are already implementing certain anchor activity projects which have an impact -though not yet measurable- on their local communities. To address further the point you raise here, we have edited our study weaknesses section by adding the following sentence on p. 14, lines 36 & p.15, lines 1-2.
"Therefore, we are confident that our conclusions are robust and meaningful for researchers and practitioners highlighting the potential that health care organisations have to operate as anchor institutions".
• We also added the following sentence in the conclusion in the abstract.
"Health care organisations have the potential for a positive impact on the overall wellbeing of local communities."
The article could be made more useful if there was a table suggesting how impact could be assessed in future.
• Thank you for your comment. Instead of offering a table we have addressed this point in the section where we discuss recommendations for future research. We have added the following sentence, on p.15, lines 33-35.
"Existing measurement toolkits are already offering a range of useful indicators for measuring this impact. Future research can help us elaborate and refine such toolkits."
• We considered that linking our work with existing tools would be more useful and consistent for researchers and practitioners and would strengthen the coherence of the evidence base of anchor activity of health care organisations which is still at an early stage at least in the UK.
The objective of the study are rather vague at the beginning of the paper (page 4, line 50) and in the abstract. The objective- a study of the mechanisms by which health care organisations can impact social determinants and local communities- should be made clearer in the abstract, in the introduction and throughout the paper.
• Thank you for your comment. We have now made the objectives clearer in the abstract and the introduction following your recommendations.
A more critical engagement with the literature on the processes of health care re-design is also needed. The present processes of transformation of the UK health care system have been interpreted in different ways and it is important to acknowledge the contested nature of these changes. The interpretation of the present changes adopted by the study is one amongst many and this should be acknowledged.

• Thank you for your comment. We have added the following text in the strengths and weaknesses section, p.14, lines 29-31.
“Our study draws on system redesign and organisational transformation in a UK setting and therefore may have limited generalisabilty in health care settings with different levers for organisational change.”

More detail of the data-driven approach to the selection of the case studies is needed. What did this approach contribute to the analysis and how are the cases chosen by this method related to the case studies located in areas of social and economic disadvantage.
• We aimed to select illustrative case studies (as per realist methods), rather than representative case studies. To clarify, we have now added the following text in the recruitment of case study sites section, p.6, lines 5-12.
“This data driven approach ensured the non-biased selection of case studies from a pool of health care organisations that were diverse across a series of comparable objectively measured indicators. Further, we focused on socio-economically disadvantaged areas because health care organisations in those areas serve the people most severely affected by health inequalities. We considered that this might imply a greater engagement with anchor activity but also challenges resulting from the increased patient need which would ensure that our findings would be meaningful within the current challenging circumstances within the healthcare system.”

The article refers to the programme theory. Please explain this in more detail and how this helped the decision of the coding.
• This has been now elaborated in the methods section, p. 5, lines 8-17.
More detail is also necessary on the section on patient and public involvement. How many public discussions groups?, what were their composition? Where any community organisations involved? If so who did they represent? Given the poor mechanisms for public and patient involvement in the health care sector, more detail of the scale and nature of this participation is needed.
• Thank you for your comment. We have now amended the relevant section as follows (p.7, lines 7-20).
“We worked closely with a diverse Public & Community Involvement & Engagement group (PCIE) from of the Greater Manchester (GM) community. We recruited participants through our partnership with NIHR Applied Research Collaboration(ARC) for GM Public and Community Involvement, Engagement, and Participation group (PCIEP) and the respective Young People Advisory Research Group (YPAG). Our PCIE group comprised of five individuals with a diverse range of experience with healthcare services and community work. In their majority, members of our group were people from ethnic minority backgrounds. RW, AG, and SK were the main points of contact for public involvement. We held five online meetings across different stages of the study where our contributors shared insights and feedback on: the formulation of research questions, quantitatively measuring anchor activity, selection of case studies, development of interview topic guide, findings and conclusions. In addition, conversations were held on a one-to-one basis via email or online meetings. All the members of our PCIE group were reimbursed for the time they engaged with our research.”

Reviewer: 4
Dr. Francisco Reyes-Santías, Universidad de Vigo, Servicio Galego de Saude
Comments to the Author:
REVIEW REPORT FOR THE STUDY “HOW CAN HEALTH CARE ORGANISATIONS IMPROVE THE SOCIAL DETERMINANTS OF HEALTH FOR THEIR LOCAL COMMUNITIES: REALIST INFORMED CASE-STUDIES”
Journal: BMJ Open

The paper "How can health care organisations improve the social determinants of health for their local communities: Realist informed case-studies", performs a study that explore how health organisations

can act effectively as grassroots institutions to improve the social determinants of health for people in their communities.

Title and summary. The title and abstract express well the object of study, objectives, and results of the article.

Structure of the article. The contents are well organized and they adhere to the IMRaD structure. It includes a theoretical framework of the research problem, but at this point, I suggest the authors incorporate some other bibliographic references that I miss in the text:

Mulumba M, London L, Nantaba J, Ngwena C. Using Health Committees to Promote Community Participation as a Social Determinant of the Right to Health: Lessons from Uganda and South Africa. Health Hum Rights. 2018 Dec;20(2):11-17. PMID: 30568398; PMCID: PMC6293345.

Ruano AL, Friedman EA, Hill PS. Health, equity and the post-2015 agenda: raising the voices of marginalized communities. Int J Equity Health. 2014 Oct 10;13:82. doi: 10.1186/s12939-014-0082-6. PMID: 25300905; PMCID: PMC4201725.

• Thank you for your positive comments and recommendations. We have now cited the suggested studies in the section where we discuss our findings in the context of previous work (references 25 & 26).

Focusing on the opportunity of the study, it must be said that it is useful work since it covers one of the major problems resulting from a health care models.

Materials and methods.

Regarding the material and methods section, the methodology is tailored to the object of study and the objectives and is explained in a transparent manner while it has been validly applied to guarantee the results.

• Thank you for this positive feedback.

Results.

The results are significant and they are presented in an adequate and understandable way not only through narration but also with self-explained tables that are also well elaborated in terms of presentation. The results justify and relate to the objectives and methods and the results are of sufficient interest.

• Thank you.

Discussion

It would be interesting to introduce in the paper a discussion to appropriately compares the study results with other works, highlighting the main study findings. I would suggest the inclusion of three bibliographic references in the discussion section:

Ahmed, S., Chase, L.E., Wagnild, J. et al. Community health workers and health equity in low- and middle-income countries: systematic review and recommendations for policy and practice. Int J Equity Health 21, 49 (2022). https://doi.org/10.1186/s12939-021-01615-y

Andermann, A. Screening for social determinants of health in clinical care: moving from the margins to the mainstream. Public Health Rev 39, 19 (2018). https://doi.org/10.1186/s40985-018-0094-7.

• Thank you for these recommendations. Although we acknowledge the importance of the suggested studies, we have not cited them in our paper in this occasion because they focus on service delivery rather than on the ways that healthcare organisations can impact social determinants of health in their local areas as economic actors.

Bibliography

The 66.6% of the bibliography cited in the study belongs to the previous five years.

Overall, it is an interesting study and should be considered for publication in BMJ Open, once the minor revisions proposed have been resolved.
• Thank you.
Reviewer: 5
Prof. Ceu Mateus, Lancaster University
Comments to the Author:
This is an interesting paper resulting from informed case studies. the capacity for health care organisations to improve the social determinants of health for their local communities seem to exist, it would be interesting to see how it materializes. It's interesting that the working culture of the institutions was not considered as relevant for the development of anchor activities by the institutions. It would be relevant to have some demographic characteristics of the sample like gender split, mean age, and number of years in the role.
• Thank you for your feedback. We consider that what you address here is to some extent captured by what we have called “organisational ethos” in the manuscript. In that, section among others we mention that a population health approach is more effective “When led by people who appreciate the importance of strengthening communities and social value, due to lived experience” because “related projects are better tailored to local needs.” If you particularly refer to cultural diversity, this is something that we explored during the interviews but it was not addressed more than what we have currently discussed in the manuscript across the different results sections. Not every participant mentioned their years in the role but we talked with people who were less than a year and others who were more than seven years in the role. We did not add a sample characteristics table because we didn’t ask participants to share their demographic characteristics.

VERSION 2 – REVIEW

**REVIEWER**	Bludau, Heidi<br>Vanderbilt University, Medicine, Health, and Society
**REVIEW RETURNED**	07-Jun-2024

**GENERAL COMMENTS**	The revisions have made this piece much clearer. I think it's a worthwhile study and find your conclusions very interesting.

**REVIEWER**	Reyes-Santías, Francisco<br>Universidad de Vigo, Organización de Empresas e Mercadotecnia
**REVIEW RETURNED**	09-Jun-2024

**GENERAL COMMENTS**	Title and summary. The adjustment of the title is positively appreciated.<br>Theoretical framework<br>The inclusion of the suggested bibliographical references in theoretical framework is positively valued.<br>Materials and methods.<br>Regarding the material and methods section, the methodology is tailored to the object of study and the objectives and is explained in a transparent manner while it has been validly applied to guarantee the results.<br>Results.<br>The results are significant and they are presented in an adequate and understandable way not only through narration but also with self-explained tables that are also well elaborated in terms of presentation. The results justify and relate to the objectives and methods and the results are of sufficient interest.<br>Discussion.<br>The inclusion of a discussion section is appreciated.<br>Bibliography.

	The 70.83% of the bibliography cited in the study belongs to the previous five years.<br>The article is proposed for publication in BMJ Open.

**REVIEWER**	Mateus, Ceu<br>Lancaster University, Division of Health Research
**REVIEW RETURNED**	17-Jun-2024

**GENERAL COMMENTS**	This version is much improved.
